# New insights into fetal mammary gland morphogenesis: differential effects of natural and environmental estrogens

**DOI:** 10.1038/srep40806

**Published:** 2017-01-19

**Authors:** Lucia Speroni, Maria Voutilainen, Marja L. Mikkola, Skylar A. Klager, Cheryl M. Schaeberle, Carlos Sonnenschein, Ana M. Soto

**Affiliations:** 1 Dept. of Integrated Physiology and Pathobiology, 136 Harrison Avenue, Tufts University School of Medicine, Boston MA 02111 USA; 2 Developmental Biology Program, Institute of Biotechnology, University of Helsinki, Finland

## Abstract

An increased breast cancer risk during adulthood has been linked to estrogen exposure during fetal life. However, the impossibility of removing estrogens from the feto-maternal unit has hindered the testing of estrogen’s direct effect on mammary gland organogenesis. To overcome this limitation, we developed an *ex vivo* culture method of the mammary gland where the direct action of estrogens can be tested during embryonic days (E)14 to 19. Mouse mammary buds dissected at E14 and cultured for 5 days showed that estrogens directly altered fetal mammary gland development. Exposure to 0.1 pM, 10 pM, and 1 nM 17 β-estradiol (E2) resulted in monotonic inhibition of mammary buds ductal growth. In contrast, Bisphenol-A (BPA) elicited a non-monotonic response. At environmentally relevant doses (1 nM), BPA significantly increased ductal growth, as previously observed *in vivo*, while 1 μM BPA significantly inhibited ductal growth. Ductal branching followed the same pattern. This effect of BPA was blocked by Fulvestrant, a full estrogen antagonist, while the effect of estradiol was not. This method may be used to study the hormonal regulation of mammary gland development, and to test newly synthesized chemicals that are released into the environment without proper assessment of their hormonal action on critical targets like the mammary gland.

Fetal exposure to endogenous and synthetic estrogens has been linked to an increased risk of developing breast cancer^1^,^2^,^3^,^4^,^5^, yet the pathways by which estrogens alter fetal mammary gland development remain to be elucidated. In rodents, alpha-fetoprotein (AFP) present in amniotic fluid and fetal serum binds to ovarian estrogens decreasing their bioavailability thereby protecting the fetus from harmful levels of estrogen^6^. Thus, the presence of AFP hinders the study of direct effects of estrogen on the mouse fetal mammary gland. Notwithstanding, environmental estrogens such as BPA increased the propensity of developing mammary cancer^7^,^8^,^9^,^10^,^11^,^12^. The xenoestrogen BPA is widely employed in the manufacture of polycarbonate plastics and epoxy resins and it is present in products used on a daily basis^13^ such as thermal paper^14^,^15^. BPA has been detected in more than 90% of urine from samples representative of the US population suggesting that human exposure to the chemical is widespread^16^. BPA has also been detected in the blood of adults, and in the placenta, umbilical cord and fetal plasma indicating that the fetus is exposed to BPA in the womb. Perinatal exposure to BPA has been linked to the development of a plethora of metabolic^17^,^18^, behavioral^19^,^20^,^21^, and reproductive^22^ disorders. Fetal exposure to BPA alters the overall organization of the mouse mammary gland, impairs mammary gland development causing functional lactational changes^23^ and increases the risk of developing mammary cancer during adulthood^24^,^25^,^26^. At E18, the mammary glands of fetuses exposed to low dose of BPA showed accelerated adipogenesis, decreased expression of tenascin C and versican^27^, altered collagen deposition in the stroma, accelerated ductal growth and delayed lumen formation^28^. BPA exposure induced similar changes in the fetal mammary gland of non-human primates^29^. However animal models cannot reveal whether this BPA effect is mediated directly through the estrogen receptors (ER) present in the fetal mammary gland stroma^27^ and/or indirectly through the hypothalamic–pituitary–gonadal axis (HPOA)^30^.

The newly developed *ex vivo* culture method described herein makes it possible to examine the direct action of estrogens and estrogen-mimics on fetal mammary gland development. Previously we described a methodology that ensures reproducibility^31^,^32^; however, this culture technique is not suitable for the study of estrogen action since it uses culture medium containing serum and thus endogenous estrogens. We have now modified that culture method by using estrogen-depleted serum. This modification enabled us to perform a quantitative analysis of the consequences of exposure to estrogenic compounds based on morphometric parameters.

## Results

### Cultured fetal mammary glands of CD-1 mice develop in estrogen-free conditions and show similar structures as those observed *in vivo*

**Figure 1:**
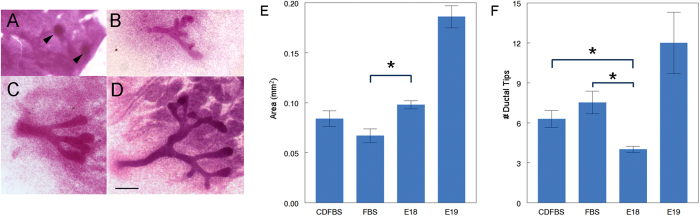
Comparison between CD-1 mouse fetal mammary glands grown *in situ* and *in culture*. Whole mounts of (**A**) E14 mammary glands, (**B**) glands cultured *ex vivo* in CDFBS for 5 days, (**C**) *in situ* embryonic mammary glands at E18 and (**D**) *in situ* embryonic mammary glands at E19. Arrows point to mammary buds. Scale bar: 200 μm. Morphometric analysis comparing fetal mammary gland development *in vivo* with cultured explants. Graphs show (**E**) area of ductal growth and (**F**) number of ductal tips. Asterisk denotes significance. Data from three independent experiments, n = 25 and 21 for CDFBS and FBS cultured mammary glands, respectively; n = 39 for E18 and n = 5 for E19, shown for comparison.

**Figure 2:**
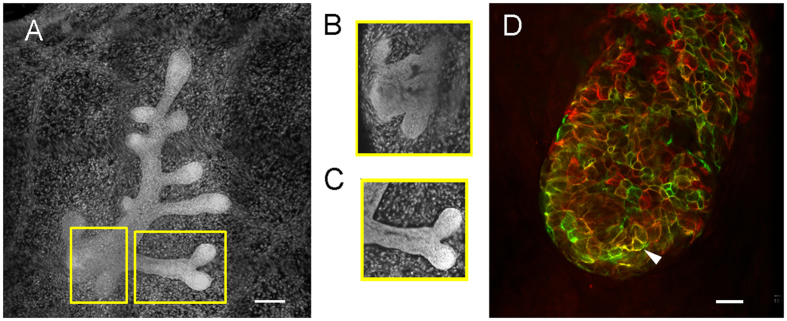
Development of the epithelium in cultured *ex vivo* mammary glands. (**A**) Confocal projection of a fetal mammary gland cultured *ex vivo* for 5 days. Scale bar: 100 μm. (**B**) and (**C**) Inset boxes from panel A are optical sections showing lumen. (**D**) Ductal tip of a cultured mammary gland shows expression of K8 (red) and K14 (green); arrowhead points to K8+/K14+ cells. Scale bar: 20 μm.

We first compared the development of the fetal mammary ductal system in hormone-free conditions [10% charcoal dextran-stripped fetal bovine serum (CDFBS)] to that obtained when the mammary glands were cultured in medium containing 10% FBS. Ductal area and number of ductal tips were measured in carmine-stained whole-mounts of the cultured mammary glands (Fig. 1). Ductal area was larger in 10% CDFBS than in 10% FBS, while the number of ductal tips, a measure of complexity, was higher in glands cultured in 10% FBS compared to 10% CDFBS, but these differences did not reach statistical significance. Ductal area in 10% CDFBS was comparable to that observed in E18 mammary glands developed *in situ* and there was a significantly higher number of ductal tips in the former than in the latter (P = 0.02). In contrast, the ductal area in 10% FBS was significantly smaller than the one in the E18 developmental stage (P < 0.0005) (Fig. 1). These findings suggest that ductal growth is inhibited to some extent by the estrogens present in FBS, which are removed by charcoal dextran-stripping. The number of ductal tips of the cultured mammary glands falls between that found in fetuses at E18 and 19. In order to further confirm that the stage of development in culture resembles the stage *in situ*, we analyzed the epithelial compartment for lumen formation and markers of mammary epithelial differentiation. After 5 days in culture, lumen formation, a feature observed *in vivo* at E18^28^,^33^, was also observed when using confocal microscopy (Fig. 2A,B and C). The mammary epithelial cells in the explant expressed cytokeratin (K) 14 and K18 (Fig. 2D), as observed *in vivo* at E18.5^34^.

### Fetal mammary glands cultured *ex vivo* respond to hormonally-active chemicals: E2 inhibits ductal development while BPA increases it at low doses and inhibits it at high doses

**Figure 3:**
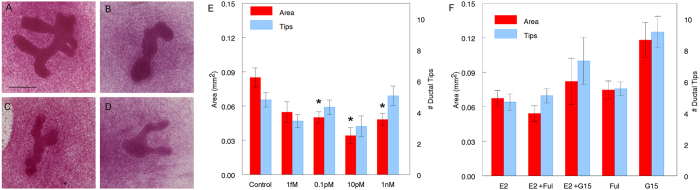
Effect of E2 on fetal mammary glands cultured *ex vivo*. Carmine stained whole mounts of cultured mammary explants in (**A**) Control (CDFBS), (**B**) 1 nM E2, (**C**) 10 pM E2 and (**D**) 10 pM E2 + 10 nM Ful. Scale bar: 200 μm. (**E**) Ductal area and number of ductal tips of mammary buds treated with E2 compared to control. Asterisk denotes significance compared with control. Data from three independent experiments, n = 18 for area and n = 17 for number of ductal tips in control, n = 9 in 1 fM, n = 14 for area and n = 15 for number of ductal tips in 0.1 pM, n = 9 for area and n = 10 for number of ductal tips in 10 pM and n = 16 in 1 nM group. (**F**) Effect of Ful (10 nM) and G-15 (1 nM) on E2 (10 pM) -treated mammary bud cultures. Data from three independent experiments, n = 21 for area and n = 20 for number of ductal tips in E2, n = 17 for area and n = 16 for number of ductal tips in Ful, n = 12 for area and n = 11 for number of ductal tips in G-15, n = 19 for area and n = 18 for number of ductal tips in E2 + Ful and n = 9 in E2 + G15.

**Figure 4:**
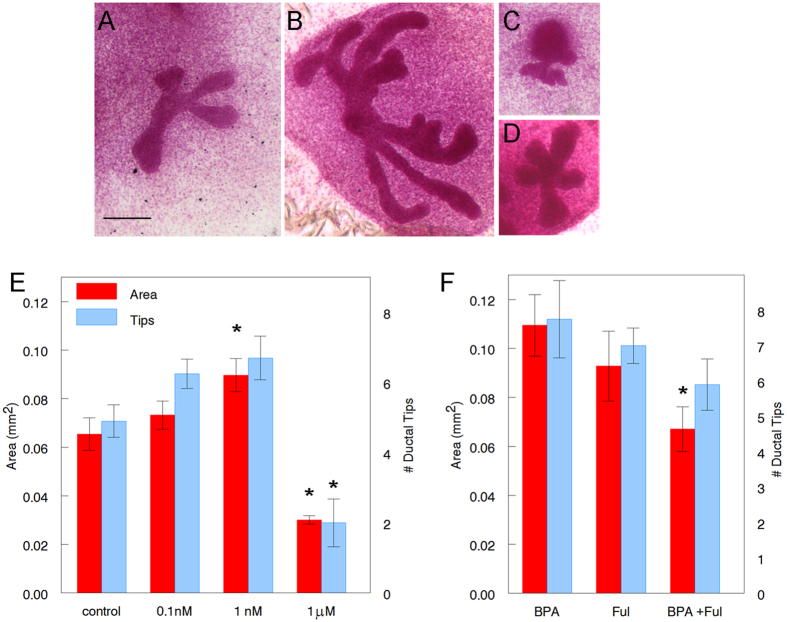
Effect of BPA on fetal mammary glands cultured *ex vivo*. Carmine stained whole mounts of cultured mammary explants in (**A**) Control (CDFBS), (**B**) 1 nM BPA, (**C**) 1 μM BPA and (**D**) 1 nM BPA + 100 nM Ful. Scale bar: 200 μm. (**E**) Ductal area and number of ductal tips of mammary buds treated with BPA compared to control. Asterisk denotes significance compared with control. Data from three independent experiments, n = 22 for area and n = 21 for number of ductal tips in control, n = 12 in 0.1 nM, n = 20 in 1 nM and n = 8 for area and n = 9 for number of ductal tips in 1 μM group. (**F**) Effect of Ful (100 nM) on BPA (1 nM) -treated mammary bud cultures. Asterisk denotes a statistically significant difference from BPA alone. Data from three independent experiments, n = 9 for area and n = 8 for number of ductal tips in BPA and in Ful and n = 10 in BPA + Ful.

The cultured mammary glands were exposed to a range of E2 (Fig. 3) and BPA (Fig. 4) concentrations for a period of 5 days. Cultures were harvested, whole-mounted and stained with carmine-alum and assessed by morphometric analysis. Epithelial growth was significantly diminished in mammary buds exposed to 0.1 pM, 10 pM, and 1 nM E2 compared to control (P = 0.005, <0.0005 and =0.002, respectively); while 1 fM E2 had no effect (Fig. 3E). Exposure of the explants to 1 nM BPA significantly increased ductal growth (P = 0.032) while 1 μM BPA significantly decreased it (P = 0.016) when compared to control (Fig. 4E), showing a non-monotonic dose-response curve. This is consistent with previous results obtained *in vivo* where increased ductal development was observed in fetuses of dams exposed to low BPA doses^28^. The number of ductal tips was significantly decreased in explants exposed to 1 μM BPA (P = 0.012) when compared to control (Fig. 4E).

### Role of ER and GPER stromal receptors in the effect of E2 and BPA on ductal development

Among estrogen ligands, only ERα and β^28^,^35^ and G protein-coupled estrogen receptor 1 (GPER)^27^ are expressed in the mesenchyme of the fetal mammary gland; they are not detected in the epithelium at this developmental stage. The effect of BPA was reversed by Fulvestrant (Ful), a nuclear ER antagonist^36^ (Fig. 4D and F). The ductal area of cultured mammary glands exposed to BPA + Ful was significantly smaller than that of glands treated with BPA alone (P = 0.013) (Fig. 4F). On the contrary, the effect observed with E2 was not reversed by treatment with Ful (Fig. 3D and F). The ductal area and number of ductal tips of mammary glands treated with E2 + Ful was similar to that of E2 alone (Fig. 3F). The ductal area and number of ductal tips of the cultured mammary glands exposed to E2 + G-15, a GPER antagonist, were not significantly different from those exposed to E2 alone (Fig. 3F).

## Discussion

Fetal exposure to xenoestrogens has long-term consequences regarding the risk of developing breast cancer during adult life^37^. For example, fetal exposure to the pesticide dichloro-diphenyl-trichloroethane (DDT)^38^ and diethylstilbestrol (DES) results in a higher risk of developing breast cancer than in unexposed women^2^. Similarly, exposure to the environmental estrogen BPA, a compound structurally related to DES, has been shown to induce intraductal hyperplasias, ductal carcinoma *in situ* (DCIS) and palpable tumors in rodents^7^,^8^,^9^,^10^,^11^,^12^,^39^. Early life exposure to these estrogenic compounds is associated with the risk of developing breast cancer later in life; however the gap of several decades that exists between the time of exposure and that of the clinical detection of neoplasia makes it difficult to identify the chain of events that lead to breast cancer. In addition to the indirect effect of these estrogens on the HPOA, we have hypothesized that those initial causal events occur through a direct action of the estrogenic compounds on mammary gland morphogenesis^4^. In this regard, using our culture method, we found that environmentally relevant doses of BPA increased ductal development in a comparable manner to that observed in the fetal mammary glands of mice and primates exposed *in utero*^28^,^29^, whereas high doses inhibited it.

*In utero* exposure to natural estrogen has also been associated with an increased risk of developing breast cancer in twin pregnancies^40^ and a decreased risk associated with pre-eclampsia^41^. These correlations assume that two placentas represent higher estrogen exposure than a single one, and that pre-eclampsia is a marker of low estrogen levels. Due to the impossibility of removing estrogens during pregnancy, there is a lack of experimental evidence of the action of E2 on the fetal mammary gland. Reports in the literature regarding the effect of exogenous E2 on the developing mammary gland used supra-physiological doses. One examined E18 mammary glands of female mice embryos injected *in utero* and found inhibition of mammary development beyond the bud stage^42^. Another report, now in humans, showed that still-born fetuses whose mothers were treated with estrogen and progesterone during early pregnancy also exhibited inhibition of mammary gland development^43^. In contrast, by exposing the explants to physiological levels of estradiol we found that these levels do not arrest mammary gland development at the bud stage; instead, it resulted in the development of an epithelial tree smaller than that of the unexposed control.

We have also gained insight into how BPA and E2 affect the fetal mammary gland. The effect of BPA was inhibited by the antiestrogen Ful suggesting that BPA is acting through nuclear ERs present in the stroma^27^,^28^,^44^. In contrast, the effect of E2 was not inhibited by Ful, suggesting that at this early stage of development E2 could be acting through an alternative pathway. By revealing functional differences between the effect of E2 and BPA, our findings illustrate the complexity of hormone regulation in target organs. This complexity was also reflected by the gene expression pattern of fetal mammary glands exposed to different estrogens. The gene expression pattern of the stroma of BPA- exposed animals mostly overlapped with that of the stroma exposed to the steroidal estrogen ethinyl estradiol. However, the patterns were not identical, showing a set of genes that were differentially regulated by each one of these estrogens^27^. A comparable result was obtained when using estrogen-sensitive MCF7 cells^45^. In addition to gene expression end points, functional differences between BPA and estradiol have also been reported in the brain where E2 causes a rapid stimulatory action in luteinizing hormone-releasing hormone neurons and this effect is not blocked by Ful^46^,^47^; this suggests that the action of E2 is independent of ER and in fact it appears to be mediated by GPER^48^. We initially hypothesized that E2 could be acting through GPER present in the stroma of the fetal mammary gland^27^. The effect of E2 was not reversed by treatment with G-15, a GPER antagonist. However, the role of GPER cannot be excluded at this point due to the variability observed in the mammary explants treated with G-15. The use of the *ex vivo* culture system presented herein will allow us to further explore the manner by which E2 inhibits ductal growth, for example by using specific antagonists for ERα and ERβ and receptor null mutants.

Finally, the *ex vivo* explant culture and the morphological measurements described herein as indicators of altered mammary gland development could be used as reliable tools to assess compounds for their likelihood to contribute to altered mammogenesis, which in turn is a prerequisite for neoplasia.

## Conclusion

By using a unique *ex vivo* culture assay that circumvents the impossibility of removing estrogens from the feto-maternal unit, we show that natural and environmental estrogens directly alter fetal mammary gland development. The alterations observed occur within physiological levels of estradiol and of environmental BPA exposure. Additionally, this *ex vivo* culture assay makes it possible to examine the developmental toxicity of environmental chemicals suspected to cause breast cancer. This study also provides further insight into a) the role of estrogens and xenoestrogens on breast carcinogenesis, and b) the action of hormones on normal mammary gland development.

## Materials and Methods

### Dissection and culture of CD-1 fetal mammary glands

CD-1 mice were purchased from Charles River and maintained at the Tufts University School of Medicine animal facility. All animal procedures were approved by the Tufts University and Tufts Medical Center Institutional Animal Care and Use Committee (Animal Welfare Assurance no. A3775-01, protocol number B2013-138) in accordance with the Guide for Care and Use of Laboratory Animals. The methods carried out in this work are in accordance with approved guidelines. CD-1 mice were mated and females were checked for the presence of vaginal plugs. The morning that the vaginal plug was observed was considered E1. Fetuses were removed from the pregnant mouse at E14. The mammary buds of female embryos were dissected and cultured following the technique described by Voutilainen *et al*.^31^,^32^. The mammary explant typically contained mammary buds #2, 3, and 4. Culture media was phenol red free-DMEM/F12 supplemented with 2 mM L-glutamine, 10% CDFBS (except for cultures using FBS), 75 μg/ml ascorbic acid and penicillin/streptomycin (base media). For details on CDFBS procedures and filter membranes see Supporting Information. Explants were cultured for 5 days and culture media was changed twice during the culture period. Mammary gland development can be followed by phase-contrast microscopy (Fig. S1). After harvest, explants were left on filters during further processing. A detailed protocol of tissue dissection and culture is provided as Supporting Information.

### BPA, E2, Fulvestrant and G-15 treatments

E2 was dissolved in ethanol to obtain a 1 mM stock solution. BPA, Fulvestrant (ICI 182,780) and G-15 were dissolved in DMSO to obtain a 1 mM, 10 mM and 50 mM stock solution, respectively. Fulvestrant was used at 10 and 100 nM. G-15 was used at 1 nM. Stocks were diluted in base medium as described above to final concentrations. Control explants were cultured in base medium alone.

### Sample processing for whole mounts

On day 5 of culture, the explants were fixed in 10% paraformaldehyde and processed for carmine staining^32^. After carmine staining, explants were dehydrated using a series of ethanol dilutions (25, 50, 70 and 100%) and xylene. The explants were then whole-mounted on glass slides using permount.

### Morphometric analysis

Whole mounted explants were viewed through a Zeiss Stemi 2000-c dissection scope at 5x. Images were captured by an AxioCam HRc digital camera (Zeiss) and processed with Axiovision software (version 4.3; Zeiss). The area of the ductal growth was measured by outlining the ducts using the outline spline feature and ductal tips were counted. The most developed mammary bud in each explant according to area of ductal growth was included in the analysis. Whole mounted fourth inguinal mammary glands of E18 and E19 female CD-1 mice were imaged and measured as described for the whole mounted explants.

### Immunofluorescence staining on whole-mounts

Explants were harvested and transferred to wells of a 24-well plate. The protocol for immunostaining was adapted from Kogata N & Howard BA^44^. Briefly, explants were fixed in 4% paraformaldehyde for 1 h, blocked and permeabilized for 1 h at room temperature. The explants were incubated with primary antibodies overnight and then with secondary antibodies for 4 h at 4 °C. Primary antibodies used were cytokeratins (K) 8 (rat, 1:100) (Troma-I, Developmental Studies Hybridoma Bank) and K14 (rabbit, 1:200) (RB-9020, Thermo Fisher). The secondary antibodies (1:1000) used were Cy2-conjugated donkey anti-rat and RRX-conjugated donkey anti-rabbit (Jackson Immunoresearch). Images were acquired using a Zeiss LSM510 confocal microscope.

### Statistical analysis

SPSS software package 15.0 (SPSS) was used for all statistical analyses. The analysis was performed on data after mathematical outliers (values that fell below or above more than 2 SD from the mean) were removed. T-tests were used to compare i) the growth of cultured mammary glands with E18 mammary glands; ii) cultures exposed to E2, with E2 + G-15 and E2 + Ful, and iii) cultures exposed to BPA with BPA + Ful. One-way ANOVA and Bonferroni’s *post hoc* test was used to assess differences between controls and cultures exposed to different concentrations of E2 and BPA. For all statistical tests, results were considered significant at P < 0.05. All results are presented as mean ± SEM; n value reported applies to both ductal area and number of tips unless otherwise stated.

## Additional Information

**How to cite this article:** Speroni, L. *et al*. New insights into fetal mammary gland morphogenesis: differential effects of natural and environmental estrogens. *Sci. Rep.* **7**, 40806; doi: 10.1038/srep40806 (2017).

**Publisher's note:** Springer Nature remains neutral with regard to jurisdictional claims in published maps and institutional affiliations.

## Supplementary Material

Supplementary Information
