# Supplementary material for: New insights into fetal mammary gland morphogenesis: differential effects of natural and environmental estrogens
Source: Sci Rep. 2017 Jan 19;7:40806. doi: 10.1038/srep40806 (PMC5244390; doi:10.1038/srep40806)
Supplement: Supplementary Information [file srep40806-s1.pdf]

New insights into fetal mammary gland morphogenesis: differential effects of natural and environmental estrogens.

Lucia Speroni, Maria Voutilainen, Marja L. Mikkola, Skylar A. Klager, Cheryl M. Schaeberle, Carlos Sonnenschein, Ana M. Soto.

SUPPORTING INFORMATION

Supplemental Figure S1

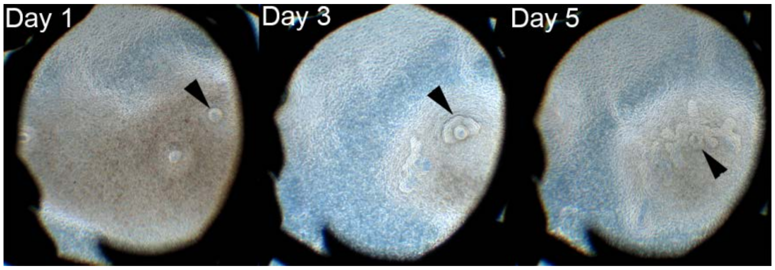

Fig S1. Time course of development of cultured mammary glands in 10% CDFBS. Phase-contrast images of live cultures. Arrowheads point to developing buds. On the first day of culture, the mammary buds show a characteristic rounded shape. By day 3 the buds have already sprouted and the mesenchyme has expanded. At the end of the fifth day of culture, the mammary glands show branches and several ductal tips.

Supplemental Text

Tissue dissection

The procedure is carried out in a laminar flow hood with a dissection stereoscope. Surgical instruments are sterilized using a glass-bead sterilizer. The mouse uterus is placed in a 10-cm

diameter glass Petri dish containing Dulbecco's Phosphate Buffered Saline (DPBS) without calcium chloride and magnesium chloride. The embryos are removed by cutting open the uterine wall using scissors and forceps, and removed from fetal membranes under a stereomicroscope. They are then transferred to a glass Petri dish with fresh DPBS, decapitated using two needles with syringes, and kept on ice. Three to four embryos at a time are transferred to a silicon dish with DPBS and processed one at a time. The embryo is attached to the silicon firmly with two needles placed on the neck and above the tail, so that the ventral side is facing upwards. The limbs are removed with scissors. Small scissors are slipped underneath the skin at the site where the hind limb has been cut off, without penetrating the underlying tissues as this will result in difficulties in subsequent dissection. Incisions are performed as depicted in [1] first incising the skin from above the hind limb and continuing along the flank of the embryo towards the forelimb. The skin is carefully detached from the embryo from the flank towards the midline using one tip of watchmaker's forceps in a horizontal sawing motion. Once the skin is loose everywhere except close to the midline (on top of the liver), the tissue explant is detached using small scissors. At this point, gonads are checked for sex identification and males are discarded. Once all the embryos have been processed, the explants are transferred to a 35-mm plastic Petri dish with small amount of DPBS using a Pasteur pipette. ***Epithelial-mesenchymal separation.*** DPBS is removed and pancreatin-trypsin working solution is added to the dish. The explants are incubated for 5 to 7 minutes at room temperature. The tissues are monitored under a stereoscope during the treatment to determine the ideal incubation time. Close to the optimal time period, the epithelium starts to release from the edges of the tissue explant. To aid the separation process, tissues are flushed slowly and gently with a Pasteur pipette and then by cautiously shaking the Petri dish during the incubation time. Pancreatin-trypsin solution is removed and DMEM-

GlutaMAX supplemented with 10 % (vol/vol) FBS is added to inactivate the enzymes. The explants should be incubated on ice for 45-50 min. After incubation, the following steps are carried out in fresh DMEM-GlutaMAX with the same supplements as above. The explants are transferred to a small glass Petri dish using a Pasteur pipette and gently flushed for 10 times. The epidermal epithelium is microsurgically removed using two needles with syringes. A needle is slipped in between the epithelium and mesenchyme, lifting the epithelium enough to be peeled off with the other needle.

Tissue Culture

Nuclepore track-etch membranes pore size 0.1 µm (Whatman Cat # 110605) are used to culture the explants. To use the membranes in tissue culture, they are washed three times in PBS and cut into small squares of desired diameter in PBS by using scissors and watchmaker's forceps. Previous to this, the filters were stored in 70% ethanol overnight. Each explant is placed on top of a filter using watchmaker's forceps. The mesenchymal side of the explant faces the filter. The filter with the explant on top is then transferred to the top of a metal grid placed inside a culture dish which was previously prepared containing 1.5-2ml of F-12/DMEM culture medium containing the hormones/chemicals and 10% CDFBS freshly supplemented with 75µg/ml ascorbic acid. The tissues are cultured in an atmosphere of 5 % CO_2 at 37 °C. The medium is changed every second day, while avoiding trapping any air bubbles underneath the filter. The explants are harvested after 5 days.

Preparation of enzyme stock and working solution

Pancreatin 10X stock solution. 2.5 g Pancreatin (Sigma, cat. no. P3292) and 0.85 g NaCl are dissolved into 100 ml of MilliQ water on a magnetic stirrer on ice for 3–4 h (or at 4 °C o/n), and the solution is centrifuged at 5000 rpm for 10 min and filter-sterilized with 0.22 μm (Millipore Stericap Plus, cat.no. SCGPCARE). The solution is then aliquoted and frozen at −20 °C; the aliquots can be stored for 1 year.

Pancreatin-trypsin working solution, pH 7.4. 0.225 g trypsin are dissolved into 6-ml Thyrode's solution on ice using a magnetic stirrer, 1 ml of 10X pancreatin stock solution and 20 μl of Penicillin-Streptomycin are added to the trypsin solution; the pH is adjusted to 7.4 with NaOH and Thyrode's solution is added to a final volume of 10 ml and sterilized by filtration through 0.22 μm filters. The solution is aliquoted into single-use aliquots which are stored at −20 °C. The pancreatin-trypsin working solution is stable for 2 weeks at −20 °C.

CDFBS

We performed an experiment to determine the mildest conditions for estrogen removal using various charcoal-dextran concentrations and changing the incubation time. We tested the resulting serum in the E-SCREEN assay. The mildest effective condition was incubation for 30 min at 37°C using 0.5% charcoal-0.05% Dextran T70 in acid washed glass roller bottles.

Estrogenic activity of filter membranes

To test the Nuclepore track-etch membranes for estrogenic activity, 10 membranes were incubated overnight in 50 ml tubes containing 70% ethanol as they are processed for the explant culture. The membranes were then extracted by transferring them to an acid-washed glass tube containing 30 ml of ethanol and incubated in a shaker at 37°C for 1 hour. The membranes were then removed and the ethanol dried under nitrogen to a final volume of 1 ml. A volume of 100 µl was used to prepare serial dilutions in phenol red-free DMEM/F12 culture medium containing 5% CDFBS and tested using the E-SCREEN assay. Estrogenicity was below the levels of quantification.

Supplemental Reference List

1 Voutilainen, M., Lindfors, P. H., & Mikkola, M. L. Protocol: ex vivo culture of mouse embryonic mammary buds. J. Mammary Gland Biol. Neoplasia **18** , 239-245 (2013).
